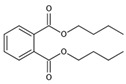# Study on Degradation of 1,2,4-TrCB by Sugarcane Cellulose-TiO_2_ Carrier in an Intimate Coupling of Photocatalysis and Biodegradation System

**DOI:** 10.3390/polym14214774

**Published:** 2022-11-07

**Authors:** Zhenqi Zhou, Chunlin Jiao, Yinna Liang, Ang Du, Jiaming Zhang, Jianhua Xiong, Guoning Chen, Hongxiang Zhu, Lihai Lu

**Affiliations:** 1 School of Resources, Environment and Materials, Guangxi University, Nanning 530004, China; 2 College of Light Industry and Food Engineering, Guangxi University, Nanning 530004, China; 3 Guangxi Bossco Environmental Protection Technology Co., Ltd., Nanning 530007, China; 4 Guangxi Key Laboratory of Clean Pulp & Papermaking and Pollution Control, Nanning 530004, China

**Keywords:** sugarcane cellulose, photocatalysis, microorganism, ICPB, 1,2,4-TrCB

## Abstract

1,2,4 trichlorobenzene (1,2,4-TrCB) is a persistent organic pollutant with chemical stability, biological toxicity, and durability, which has a significant adverse impact on the ecological environment and human health. In order to solve the pollution problem, bagasse cellulose is used as the basic framework and nano TiO_2_ is used as the photocatalyst to prepare composite carriers with excellent performance. Based on this, an intimate coupling of photocatalysis and biodegradation (ICPB) system combining photocatalysis and microorganisms is constructed. We use the combined technology for the first time to deal with the pollution problem of 1,2,4-TrCB. The biofilm in the composite carrier can decompose the photocatalytic products so that the removal rate of 1,2,4-TrCB is 68.01%, which is 14.81% higher than those of biodegradation or photocatalysis alone, and the mineralization rate is 50.30%, which is 11.50% higher than that of photocatalysis alone. The degradation pathways and mechanisms of 1,2,4-TrCB are explored, which provide a theoretical basis and potential application for the efficient degradation of 1,2,4-TrCB and other refractory organics by the ICPB system.

## 1. Introduction

1,2,4 trichlorobenzene(1,2,4-TrCB) is a typical persistent organic pollutant with characteristic stability and biological toxicity that degrades slowly in its natural state. It is widely found in soil, groundwater, wastewater, and agricultural crops and easily spreads in the environment, which can cause serious harm to the ecological environment and human health [1,2]. Physical and chemical treatments for 1,2,4-TrCB contamination include adsorption, pyrolysis, oxidation, and electrochemistry. However, there are still some deficiencies such as incomplete degradation, secondary pollution, and requirements for operating costs and conditions [3]. Although the microbial degradation of organic pollutants is simple, economical, and free of secondary pollutants, its efficiency in the degradation process is limited [4,5]. At present, there are many studies on single repair techniques for 1,2,4-TrCB, but some defects exist and studies on combinations of multiple techniques are only preliminary [6].

Pollution by 1,2,4-TrCB urgently needs to be effectively solved to improve the ecological environment and alleviate the risk to human health. Therefore, for 1,2,4-TrCB, this paper uses a joint technology to fix the problem, hoping to explore a practical and effective remediation technology, which is of great significance for solving the pollution problem of refractory organic matter such as this pollutant.

The intimate coupling of photocatalysis and biodegradation (ICPB), a new method of water pollution control introduced by Rittman et al. [7] in 2008, combines photocatalytic oxidation and microbial degradation into one reactor and ensures that both are involved simultaneously in the degradation of pollutants. This method has shown superior degradation and mineralization efficiency for persistent pollutants such as phenol [8], tetracycline [9], and chlorophenol [10]. The practical application of ICPB is limited by a low bacterial load density and low microbial activity. At the same time, it also still has issues in terms of reliability, economy, and universality of actual wastewater treatment plants so this technology has not been applied in practice [11,12]. However, the related research is extensive and in-depth and it is believed that ICPB technology has good application potential.

At present, the composite carriers used in the ICPB system include polyurethane sponge carriers, porous ceramics, and cellulose. Because of its low density, polyurethane sponge is conducive to uniform diffusion with water flow and has good adsorbability, but it is difficult to maintain the stability of biofilm after repeated use [13]. Compared with polyurethane sponge carriers, ceramic carriers can maintain the stability of biofilm even after repeated use. However, due to its high density, it is difficult to ensure that it can operate with water flow so the system’s efficiency cannot be guaranteed [14]. Cellulose has excellent adsorbability and can immobilize catalysts and biofilm. At the same time, it is a bio-friendly material and will not produce secondary pollution.

Therefore, in this paper, sugarcane cellulose was used as the basic raw material to prepare an environmentally friendly photocatalyst carrier. The ICPB system was used for the first time in the degradation of 1,2,4-TrCB, which broadened the combined technology to the treatment of this pollutant. We investigated 1,2,4-TrCB degradation by comparing the ICPB with photocatalysis and biodegradation individually. Free radical capture experiments were carried out and the possible degradation pathway and responses to the biofilm were analyzed. The dominant microorganisms degrading 1,2,4-TrCB were investigated to illuminate the mechanism of the ICPB, providing a possible theoretical basis and practical method for the efficient degradation of 1,2,4-TrCB and other refractory organic pollutants.

## 2. Material and Methods

### 2.1. Preparing Materials

**Figure 1:**
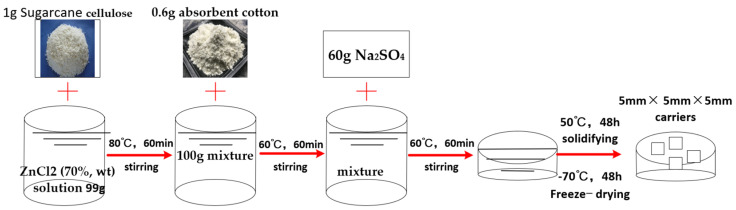
Preparation of carrier.

A cellulose carrier was prepared of bagasse cellulose, absorbent cotton, and sodium sulfate (Na_2_SO_4_) in a zinc chloride (ZnCl_2_) solution. After it was solidified in deionized water and freeze-dried, a carrier was obtained with a large number of pores. The specific process was as follows: (1) An amount of 1 g of cellulose was added to a 99 g ZnCl_2_ (70%, wt) solution and after mixing, the mixture (100 g) was stirred at 80 °C for 60 min; (2) Then, 0.6 g absorbent cotton was added into the mixture while stirring for 60 min at 60 °C, followed by the addition of 60 g Na_2_SO_4_; (3) After stirring for 60 min at 60 °C, the mixture was solidified in deionized water for 2 days and freeze-dried for 2 days at −70 °C. The carriers (5 mm × 5 mm × 5 mm) were obtained. This process is shown in Figure 1.

**Figure 2:**
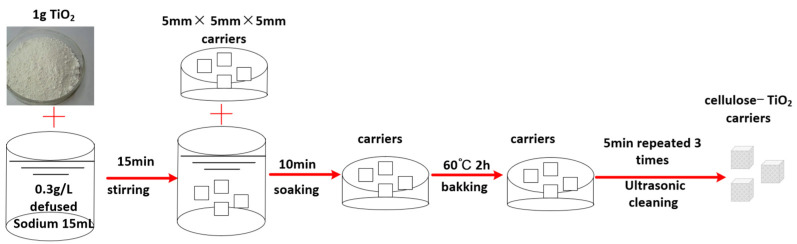
Preparation of cellulose-TiO_2_ carrier.

The catalyst was loaded onto the carriers via a simple and efficient low-temperature process, which has been previously described. In short, the process was as follows: (1) An amount of 1.5 g of visible light-responsive titanium dioxide (TiO_2_) was dissolved in a 15 mL solution of 0.3 g/L defused sodium and the mixture was stirred for 15 min to fully disperse the TiO_2_; (2) The prepared cellulose carriers were soaked in the mixed solution for 10 min to fully load the TiO_2_ photocatalyst onto the carriers; (3) The carriers were removed and baked at 60 °C for 120 min and then ultrasonically cleaned in deionized water for 5 min, and this process was repeated 3 times. This process is shown in Figure 2. The final sugarcane cellulose-TiO_2_ carrier had been prepared.

Sugarcane cellulose was obtained from Guangxi Guigang Guitang Co., Ltd., (Guigang, China) and the catalyst (visible light-responsive titanium dioxide) from Liuzhou Rose Nanomaterials Technology Co., Ltd (Liuzhou, China). Zinc chloride was purchased from Tianjin Ou Boke Chemical Products Sales Co., Ltd (Tianjin, China). Sodium sulfate was purchased from Guangdong Guanghua Sci-Tech Co, Ltd., (Shantou, China) and absorbent cotton from Nanchang Leiyi Medical Appliance Co., Ltd (Nanchang, China). Rhodamine B was purchased from Aladdin Reagent Co., Ltd(Shanghai, China). 1,2,4-TrCB was purchased from Macklin. All the chemicals were analytically pure. Activated sludge was derived from the research center of the Guangxi Bossco Environmental Protection Technology Co. in Guangxi province (Nanning, China).

### 2.2. Methods

(1) Experimental methods

**Figure 3:**
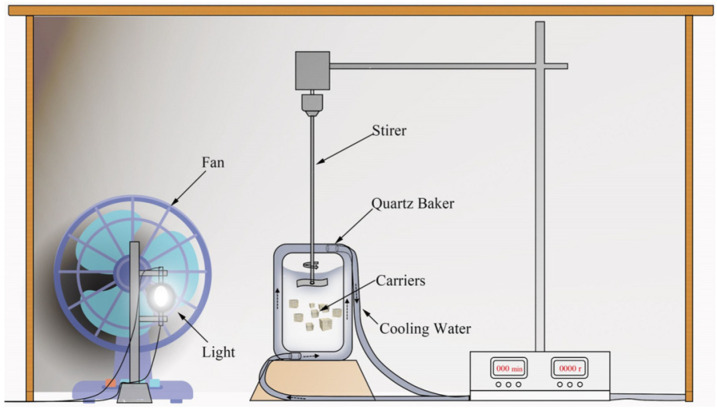
Schematic diagram of equipment.

The schematic diagram of the ICPB system is shown in Figure 3. A xenon lamp (XHA250W) was used as the light source set 15 cm away from the quartz baker (500 mL), and a stirrer was used to power the solution at 100 r/min. The initial pH was 5 and the concentrations of 1,2,4-TrCB and dissolved oxygen were 8.0 mg/L and 6.0–7.0 mg/L, respectively. The volume proportion of solution to carriers was 8%. To assess the ICPB degradation ability, the protocols, including photolysis, adsorption, biodegradation, and photocatalysis, were performed at room temperature (~26 °C).

(2) Analytical methods

The concentrations of 1,2,4-TrCB and the intermediate products were determined by a gas chromatography–mass spectrometer (5975C GC/MSD, Agilent). N-hexane was the extraction agent for the 1,2,4-TrCB and intermediate products. The initial temperature was 50 °C (maintained for 1 min), which was increased in 20 °C/min increments to 200 °C (maintained for 5 min), followed by a 10 °C/min increase to 280 °C (maintained for 1 min). The carrier gas was helium and the column flow rate was 1.0 mL/min. The injector temperature was 250 °C and the detector temperature was 280 °C. The sample of the intermediate products was obtained by concentration to reduce the liquor from 100 mL to 5 mL using rotary evaporators (RE-52A) for detection. We used sodium oxalate (1 mmol/L), tertiary butanol (1 mmol/L), and p-benzoquinone (2 mmol/L) as the trapping agents of the electron hole, hydroxyl radical, and superoxide radical, respectively [15]. A scanning electron microscope (SEM, Hitachi SU8220) was used to observe and analyze the microorganisms in the carrier before and after the experiment. The high-throughput sequencing of different samples containing the initial solution and the solution after biodegradation and ICPB was performed by Shanghai Majorbio Bio-Pharm Technology Co., Ltd. and analyzed on the cloud platform of Majorbio.

## 3. Results and Discussion

### 3.1. Degradation of 1,2,4-TrCB in ICPB

**Figure 4:**
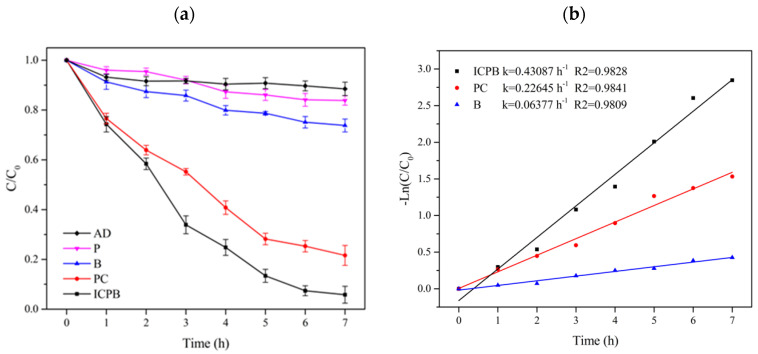
Curves of 1,2,4-TrCB degradation (**a**) and kinetics (**b**) in different protocols. AD—adsorption without light and microorganisms; P—photolysis without carriers; B—biodegradation without light; PC—photocatalysis without microorganisms; ICPB—intimate coupling of biodegradation and photocatalysis.

Figure 4 shows the degradation of 1,2,4-TrCB in different systems, referring to the reaction processes of adsorption, photodegradation, photocatalytic oxidation, and biodegradation, in addition to those in the ICPB system. The adsorption of the TiO_2_-cellulose type carrier for 1,2,4-TrCB was 11.50% after 7 h and adsorption equilibria appeared at the first hour. Therefore, adsorption had little effect on the removal of TCB. In the photolysis experiment, the removal rate of 1,2,4-TrCB was 16.15% after 7 h, indicating that photolysis can degrade 1,2,4-TrCB with low efficiency. This is consistent with the results demonstrated by Wang et al. [16]. In addition, Kozhevnikova et al. [17] found that a 16.2% removal of 1,2,4-TrCB with an initial concentration of 18 mg/L was obtained after 7.5 h under ultraviolet radiation alone (240 W, λ = 240–320 nm). Therefore, high removal was also difficult using ultraviolet radiation alone without a catalyst [18,19]. Compared with ultraviolet radiation, the Xenon lamp used in this study had a lower ability to degrade 1,2,4-TrCB because its wavelength was similar to that of natural light, which has lower energy.

In the biodegradation system (B) under the condition of microorganisms attached to the carriers without a light source, 26.20% of the substrate was degraded, which was 10.05% higher than that of single photolysis, indicating success in the domestication of the microbial degradation of 1,2,4-TrCB and that the microorganism had a certain ability to degrade 1,2,4-TrCB, as seen in Figure 4. Brunsbach et al. [20] studied indigenous microorganisms in mud soil and found that microorganisms could degrade chlorobenzene only when 1,2,4-TrCB was mixed with low-substituted chlorobenzene. In addition, Dermietzel et al. [21] and Dong et al. [22] also observed the microorganisms’ ability to metabolize 1,2,4-TrCB. Therefore, the contribution to 1,2,4-TrCB degradation by microbial sources was found. However, the long duration and low efficiency hinder its widespread application to POPs biodegradation.

Photocatalytic oxidation, an advanced oxidation method with a robust ability to degrade pollutants, is appropriate for decomposing difficult-to-degrade pollutants such as tetracycline [23], chlorobenzene [24], and hexachlorobenzene [25]. In the photocatalysis system (PC) used in this study, the removal rate of 1,2,4-TrCB was up to 79.40% for 7 h and was 53.20% higher than that of biodegradation alone, as shown in Figure 2. It was illustrated that photocatalytic oxidation should have a strong ability to degrade 1,2,4-TrCB. During the photocatalytic reaction, a large number of active groups with strong oxidizing properties, such as pores (h^+^) and hydroxyl radicals (·OH), oxidize 1,2,4-TrCB into CO_2_ and H_2_O through chlorine atom substitution and as the ring structure opens gradually.

The removal rate of 1,2,4-TrCB in the ICPB system for 7 h reached 94.21%, which was 68.01% and 14.81% higher than that of the microorganism alone and the photocatalytic oxidation system, respectively (Figure 4). The first-order kinetic rate constant of 1,2,4-TrCB degradation in the ICPB system was 0.43087 h^−1^, which was 1.9 times and 6.7 times higher than those of photocatalytic and microbial degradation systems, respectively. Kozhevnikova et al. used composite materials to catalyze the degradation of 1,2,4-TrCB and the conversion rate was 32.6% after a reaction for 7.5 h [17]. Song et al. used activated carbon-supported microorganisms to treat 1,2,4-TrCB and the degradation rate reached 48.1% in 23 day [26]. These results indicate that the use of the ICPB composite system proposed in this paper is more effective than photocatalysis or biodegradation alone.

It can be seen that photocatalytic oxidation and microbial degradation in the ICPB system did not inhibit but rather promoted each other, showing a synergistic effect on the removal rate. Considering the ICPB system’s performance regarding the degradation of other pollutants, such as phenol [27], 4-chlorophenol [10], and 2,4,6-trichlorophenol [28], the robust ability to degrade POPs appears when closely combining photocatalytic oxidation and microbial degradation.

### 3.2. Mineralization of 1,2,4-TrCB in ICPB

**Figure 5:**
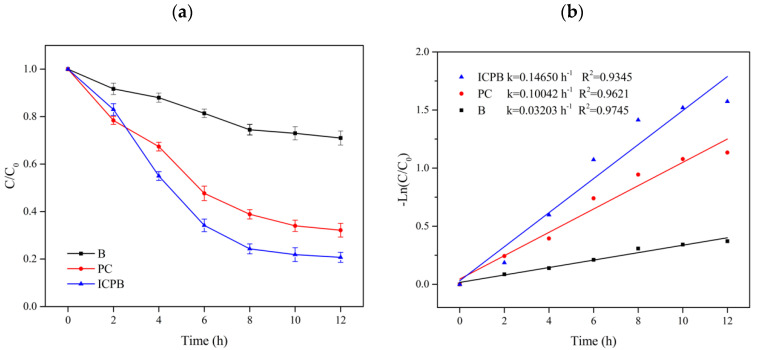
Curves of TOC degradation (**a**) and kinetics (**b**) in B, PC, and ICPB. B—biodegradation without light; PC—photocatalysis without microorganism; ICPB—intimate coupling of biodegradation and photocatalysis.

In order to reveal the mineralization effect on the ICPB system, the content of total organic carbon (TOC) was detected in the 1,2,4-TrCB degradation process using the different systems, as shown in Figure 5. Compared to the photocatalytic oxidation and coupling system, the biodegradation mineralization rate was the lowest. As expected, the power of mineralization from photocatalysis was robust and the removal rate of TOC reached up to 67.8%. However, the effect was still inferior to that of the ICPB system. Moreover, some studies have confirmed that photocatalytic oxidation may cause excessive oxidation of pollutants and does not complete mineralization, accounting for the lower rate compared to that of ICPB. The mineralization rate of 1,2,4-TrCB in the ICPB system was 79.30%, which was 11.50% and 50.30% higher than that of photocatalytic oxidation (PC) and biodegradation (B), respectively, and the first-order kinetic rate constant was increased by 0.46 times and 3.57 times, respectively. Due to the presence of microbial community in the ICPB system, the intermediate products produced by photocatalytic oxidation of 1,2,4-TrCB were relatively low in toxicity and easy to be metabolized and degraded by the microbial community, which reduced the possibility of excessive oxidation to a certain extent. At the same time, the competition between the intermediate and 1,2,4-TrCB for active species was reduced, and degradation and mineralization were promoted. This mechanism has been proved to a certain extent by Wen et al. [29] in the study on 2,4-dinitrotoluene’s degradation in the ICPB system: Although the degradation rate of 2,4-dinitrotoluene was close in both the photocatalysis and the ICPB systems, the amount of organic nitrogen transformed into NH_4_^+^-N, NO_2_-N and NO_3_-N in the ICPB system highly exceeded that of the photocatalytic oxidation. This indicates that photocatalytic oxidation has a synergistic effect on the microbial community in the system, which promoted the degradation and mineralization of 1,2,4-TrCB.

### 3.3. Construction of Free Radicals in ICPB

**Figure 6:**
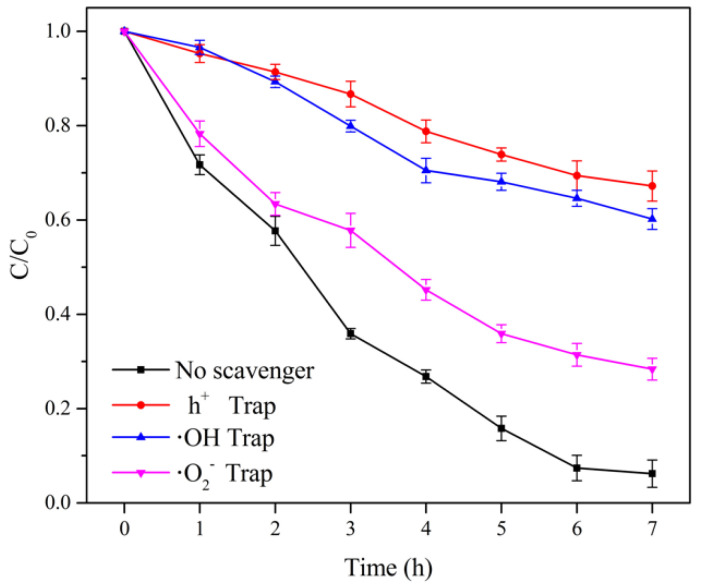
Curves of 1,2,4-TrCB with different trapping agents.

Active species with strong oxidation abilities are the major contributors to pollutant degradation. In this experiment, the effect of electron holes (h^+^), hydroxyl radicals (·OH), and superoxide radicals (·O_2_−) on the 1,2,4-TrCB removal rate was explored by adding the trapping agents of the corresponding active species, and the contribution to the different active species by the ICPB system was clearly visible, as shown in Figure 6. The removal rate of 1,2,4-TrCB in the ICPB system decreased from 93.80% to 32.72% when electron holes were trapped. The large decline of 54.46% demonstrated that electron holes play a major role in the degradation of 1,2,4-TrCB. Although they directly oxidized 1,2,4-TrCB [30], electron holes also promoted the production of hydroxyl radicals, as shown in Equations (1) and (2) [31].

Hydroxyl radicals can oxidize numerous refractory pollutants indiscriminately including 4-chlorophenol [32], hexachlorobenzene, o-dichlorobenzene, and 2,4-dichlorophenoxyacetic acid, among other pollutants. Therefore, hydroxyl radicals play a major role in a liquid-phase photocatalytic reaction [33]. In this experiment, when the capturing agent of hydroxyl radicals was added, the 1,2,4-TrCB removal rate significantly decreased. As seen in Figure 6, the removal rate of 1,2,4-TrCB decreased from 93.80% to 39.77%, indicating that hydroxyl radicals in the ICPB system also significantly contributed to 1,2,4-TrCB degradation.

(1) $$\mathrm{Catalyst}+\mathrm{hv}\to e^{-}{+\;h}^{+}$$

(2) $${\mathrm{OH}}^{-}{+\;h}^{+}\to \cdot \mathrm{OH}$$

(3) $$e^{-}{+\;O}_{2}\to \cdot O_{2}^{-}$$

As described in Equation (3) [31], during the photocatalysis process, the dissolved oxygen in the solution captured electrons and became superoxide radicals, which not only directly participated in pollutant degradation but also promoted the generation of hydroxyl radicals through the protonation process and improved photocatalytic oxidation efficiency. Lichtenberger et al. [34] found that superoxide radicals could oxidize dichlorophenol to chlorophenol. Additionally, Lin et al. [35] proved the oxidization and crack of 1,2,4-TrCB into small molecules under an attack by superoxide radicals. After adding the capture agent of superoxide radicals, the removal rate of 1,2,4-TrCB in the ICPB system decreased from 93.80% to 72.64%, demonstrating that superoxide radicals promoted the degradation of 1,2,4-TrCB, though the effect was weaker than those of hydroxyl radicals and electron holes. Considering the demands of microbial metabolism in the ICPB system, the dissolved oxygen was consumed gradually, which weakened the role of superoxide radicals in this system.

### 3.4. Degradation Pathway of 1,2,4-TrCB in ICPB

**Figure 7:**
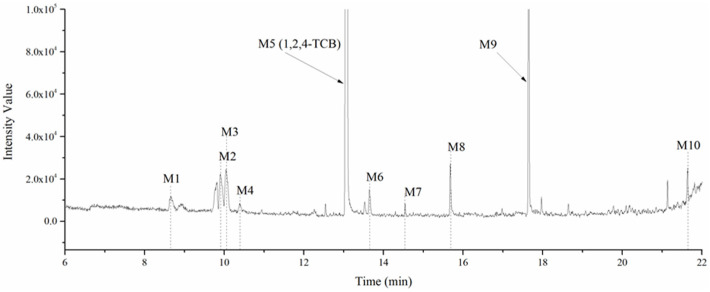
Chromatography of intermediate products of 1,2,4-TrCB degradation. The numbers M1 to M11 are the intermediate products corresponding to Table 1.

The chromatogram of the 1,2,4-TrCB degradation intermediates in the ICPB system is shown in Figure 7. There were 10 possible intermediate products, as shown in Table 1, including aromatic and chain compounds. The aromatic compounds were, respectively, C_6_H_4_Cl_2_ (o-dichlorobenzene, p-dichlorobenzene), C_11_H_10_O_6_ (3,4-bismethoxycarbonyl benzoic acid), C_14_H_22_O (2,4-di-dutylphenol), and C_16_H_22_O_4_ (1,2-dicarboxylate -dibutyl benzene) and the chain compounds included C_10_H_22_O (tetra-hydrolavandulol), C_10_H_20_O (2-decenol), C_8_H_18_O (2-ethylhexanol), and C_6_H_14_O_2_ (3-Hexyl hydroperoxide), with the functional carboxyl and hydroxyl groups. As described above, photocatalysis significantly contributed to 1,2,4-TrCB degradation, of which the reactions of substitution, addition, and ring opening occurred by the oxidization of active species and produced a series of simple organic compounds [36].

When 1,2,4-TrCB was adsorbed to the surface of the catalyst, numerous opportunities for oxidation reactions occurred between the substrates and the active groups. The experimental investigations showed that electron holes directly reacted with 1,2,4-TrCB to produce dichlorobenzene radical (·DPC) and other substances [17]. Furthermore, 1,2,4-TrCB gradually dechlorinated to produce chlorobenzene with low substituted numbers. Breaking the bond between C and Cl in the second position, first position, and fourth position in order needed progressively more energy [37]. Therefore, para-dichlorobenzene dominated the list of intermediate products, followed by m-dichlorobenzene and ortho-dichlorobenzene, respectively. However, m-dichlorobenzene could not be detected in this study for unclear reasons and should be further explored. For the low-substituted chlorobenzenes or others, chlorophenols or phenolic substances were produced through nucleophilic substitution by the attack of the hydroxyl and superoxide radicals, and even the ring structure broke into the single-stranded structure [38]. The products were further oxidized and decomposed into small molecules and further mineralized into CO_2_ and H_2_O. Considering what follows in this paper, microflora played an important role in the degradation of 1,2,4-TrCB, though the exact mineralization pathway is unknown.

**Figure 8:**
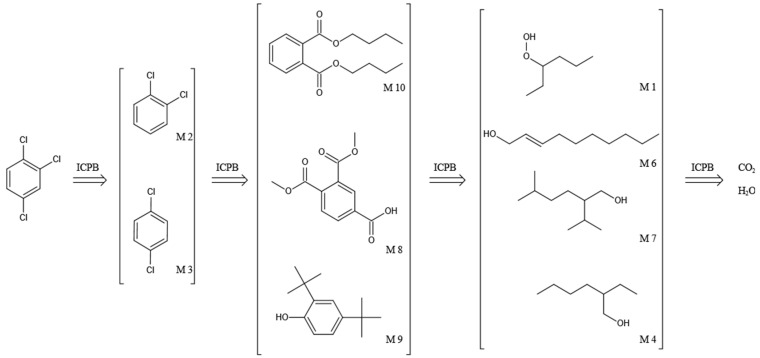
Degradation pathway of 1,2,4-TrCB.

The general degradation pathway is introduced in Figure 8. Firstly, the chlorobenzenes (M2, M3) with low substitution numbers were produced by oxidation and substitution reactions. Secondly, the chlorobenzenes were further oxidized into phenolic compounds or reacted with others to form ester compounds (M8, M9, M10). Next, the ring structures cracked into the short-chain fatty acids or alcohol (M1, M4, M6, M7) and were finally transformed into CO_2_ and H_2_O. The results were consistent with the degradation rule of 1,2,4-TrCB and other pollutants’ decompositions in the ICPB, which to some extent revealed the degradation process of 1,2,4-TrCB in the ICPB and further deepened the understanding of the operational mechanism of the ICPB system. However, further research is required on different degradation systems and the metabolic process of microorganisms to fully unravel the specific pathway.

### 3.5. Microbial Community Response Analysis

#### 3.5.1. SEM Analysis of Microorganisms in Carriers

**Figure 9:**
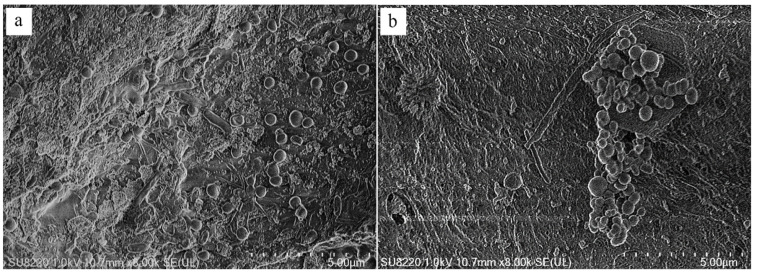
SEM images of biofilms located in carriers before (**a**) and after (**b**) ICPB.

We wanted to confirm whether the microorganisms were still stably attached to the carriers after the ICPB system had been continuously run for six cycles. Therefore, the carriers were removed for the scanning electron microscope observation in addition to the other carriers that were not included in the reaction as the control group. A random sampling of the carriers revealed abundant microorganismal growth in the carriers before and after six cycles, with a uniform distribution pattern and localized agglomeration (partial images are shown in Figure 9), indicating that the carriers protected the microorganisms from damage by the active species and the illuminant radiation. However, the distributions of biofilm in terms of quantity and distance were relatively inferior to those of the correlational studies, and a possible reason for the short one-week time for loading microorganisms was the limitations due to the experimental time. Therefore, in order to enhance the contribution to microflora, adequate time needs to be allowed.

#### 3.5.2. Genera Composition of Microbial Community

**Figure 10:**
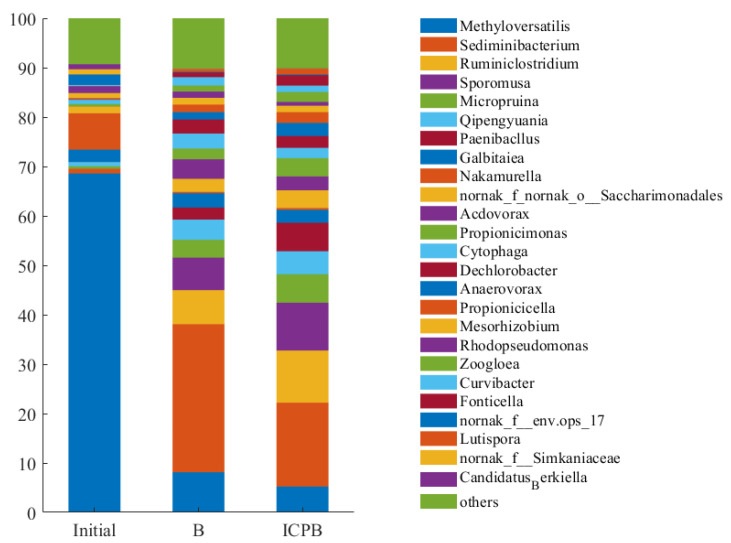
Composition of microbial communities at genus level in samples of initial, B, and ICPB. Initial—the sample before the reaction; B and ICPB—the samples of the biodegradation and coupling, respectively.

The community composition and succession are shown in Figure 10, with the relative abundance of bacteria greater than 1% at the level of genus, which includes the species of samples of the initial community, the community after biodegradation alone (B), and the community after ICPB system. Methyloversatilis genus belongs to the Proteobacteria phylum and its relative abundance decreases dramatically from the starting point to the B and ICPB systems. The results indicate that the B and ICPB systems were not conducive to the growth of the Methyloversatilis genus. Studies have shown that the Methyloversatilis genus is the dominant bacteria in the environment [39,40,41] with low-carbon organic compounds, such as methylamine, acetone, dichloroethane, etc., and the carbon sourced from the B and ICPB systems was less than that sourced from the initial system. Therefore, it was difficult to meet the metabolic demands of a large number of microorganisms and the relative abundance decreased significantly. Moreover, the Methyloversatilis genus could participate in the reduction of ClO_4_^−^ [42] and play an important role in the microbial degradation of aromatic compounds such as benzene and naphthalene [43,44], organic pesticides [45], and petroleum pollutants [46]. Therefore, the Methyloversatilis genus had a certain capacity for oxidation and chlorine resistance and could survive and contribute to 1,2,4-TrCB degradation in the B and ICPB systems. In both the B and ICPB systems, the growth of the Methyloversatilis genus was inhibited and the Sediminibacterium genus gradually became the dominant bacteria. The Sediminibacterium genus belongs to the Bacteroidota phylum and is a widespread genus in the environment ranging from soils and sediments [47] to lakes and reservoirs [48,49], mining wastewater [50], and urban rivers [51]. Hence, it plays an important role in water purification and the carbon cycle. Moreover, the Sediminibacterium genus was found to be one of the dominant bacteria in the storage pool of radioactive materials [52], and the reclaimed water was treated with a low concentration of chlorine [53], signifying its oxidation resistance. However, the dominant bacteria were replaced by the Acidovorax genus when the reclaimed water was treated with ultraviolet and chlorine together [54], indicating that ultraviolet light had adverse effects on the Sediminibacterium genus. This provides a possible explanation for why the relative abundance of this genus decreased by 13.13% in the ICPB system compared to the B system, which is that it was due to illuminant damage. In addition, the Sediminibacterium genus could use many simple and complex organic compounds, such as vinyl chloride as a carbon source necessary for growth [55,56]. Therefore, the extensive growth potential caused it to dominate in the B and ICPB systems.

The Ruminiclostridium genus, a kind of anaerobes, is commonly found in the anaerobic digestion system and can use crop stalks and other cellulose as a carbon source for anaerobic metabolism [57,58,59]. The main component of the carriers in this experiment was cellulose, which provided the conditions for the growth of the Ruminiclostridium genus. The internal structure of the carriers may be changed due to the long amount of time needed to run the ICPB system. In addition, considering the carbon source supplied to the system enhancing the metabolic activity of the microbial community, the acetic and propionic acids [60] produced by the Ruminiclostridium genus improved the degradation efficiency of 1,2,4-TrCB. However, it was not clear whether the Ruminiclostridium genus directly degraded the 1,2,4-TrCB or not. The Sporomusa genus, a kind of anaerobic bacteria belonging to Firmicutes that is widely distributed over hypoxic sediments of freshwater rivers, lakes, streams, and ditches, used a variety of electron donors for metabolism. The Sporomusa genus produced Cobamides with the functions of carbon skeleton rearrangement, methyl transfer, and reductive dechlorination [61]. A study has shown that the Sporomusa genus is conducive to the dichlorination of the Dechloromonas genus [62]. Furthermore, while degrading the pollutants, it transformed carbon dioxide into acetic acid and hydrogen, providing electron donors for other microorganisms and making a difference in the microbial community.

**Figure 11:**
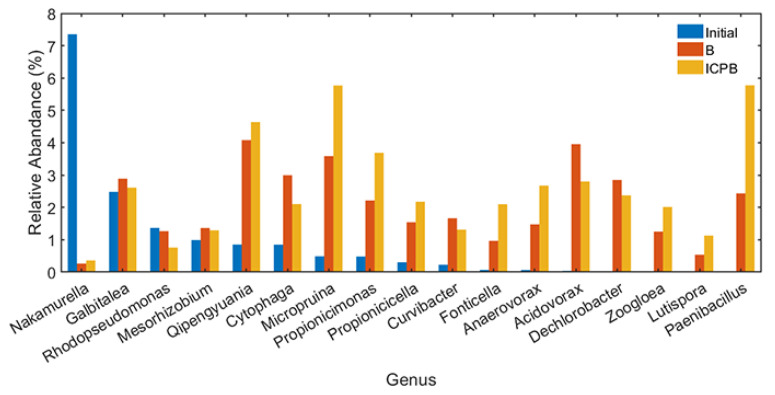
Other genera with a relative abundance of more than 1% in the samples of initial, B, and ICPB. Initial—the sample before the reaction; B and ICPB—the samples of the biodegradation and coupling, respectively.

Other bacterial genera with a relative abundance of more than 1% are shown in Figure 11. It is evident that microflora succession occurred during the degradation process of 1,2,4-TrCB. Compared with the sample of initial microbial community, a variety of genera that may be involved in the degradation of 1,2,4-TrCB were enriched in the B and ICPB systems, mainly the Micropruina genus, Qipengyuania genus, Paenibacllus genus, and Acidovorax genus. The Micropruina genus had a strong ability for total tolerance and stored lactic acid, acetic acid, and ethanol as glycogen, making a great contribution to the removal of the phosphorus and chemical oxygen demand (COD) from water bodies [63]. The Qipengyuania genus is widely distributed in freshwater, seawater, sediment, and other environments and has functional genes for the cycling of nitrogen, sulfur, and phosphorus [64]. The Qipengyuania genus has the potential to degrade microbial soluble metabolites and there are a few studies on water resource purification. The Paenibacllus genus resisted the negative influence of extreme temperatures, pH, pressure, and ultraviolet radiation due to spore formation for reproduction [65] and participated in the degradation of organic pesticides and polycyclic aromatic hydrocarbons [66]. Acidovorax, which can alleviate the oxidation of free radicals, is commonly seen in studies about the degradation of pollutants and has a significant effect on the biodegradation of aromatic compounds such as phenols, biphenyl, and chlorobenzene [67]. Therefore, the microbial community was involved in the degradation of 1,2,4-TrCB with reasonable speculation and improved the removal rate of 1,2,4-TrCB in the ICPB (Figure 6). Moreover, a functional community structure containing aerobic, facultative, and anaerobic microorganisms was constructed in the ICPB with high stability for pollutant degradation. The community structure of the biofilm in the carriers changed in the ICPB with the dominant microorganisms varying from the Methyloversatilis and Nakamurella genera to the Sediminibacterium, Ruminiclostridium, Sporomusa, and Methyloversatilis genera.

#### 3.5.3. Correlation Analysis of Microbial Community

**Figure 12:**
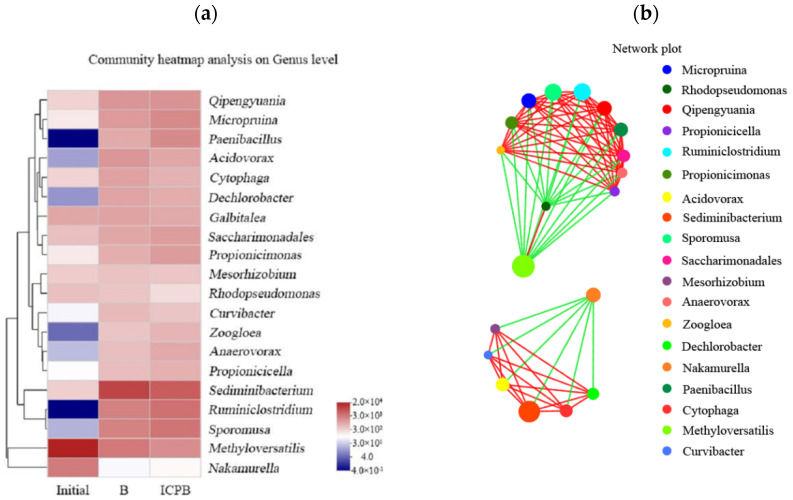
Correlation analysis of microflora. (**a**) Image of heatmap and relative abundances from low to high with colors varying from blue to red; (**b**) Network plot of the community, with each point referring to one kind of genus. The red and blue lines refer to the positive and negative correlations, respectively.

The correlation analysis of the microflora is shown in Figure 12. The community heatmap analysis shows not only the kinships of the main genera but also the relative abundances of the main genera of the different samples (initial, B, and ICPB); the color gradient from blue to red corresponds to the relative abundances from low to high. The network plot shows the correlations between the main genera; the green and red lines represent negative and positive correlations, respectively, and the nodes’ sizes represent the relative abundances. There was a significant correlation between the top 20 bacteria in the three samples (*p* < 0.05, R > 0.9). The average degree of the nodes was 9.16 and the average clustering coefficient was 1, indicating that all of the nodes were connected with the other nodes and that each genus played an important role in the community. Moreover, the microorganisms with close kinships were consistent with each other when their abundances and correlations with the others changed. The Methyloversatilis, Rhodopseudomonas, and Nakamurella genera had the same abundance variation trends for the three samples and were negatively correlated with the other bacteria connected by lines. The competitive relationship with the others could come from the three genera. Therefore, a possible kind of reference to optimize the structure of the microbial community was provided by the consistent changes in the microorganisms.

### 3.6. Degradation Mechanism of 1,2,4-TrCB in ICPB

In conclusion, considering the studies on pyridine [68], trichlorophenol [69], nitrophenol [29], and other pollutants in the ICPB system, a synergistic effect was found between adsorption, photocatalysis, and biodegradation [70]. In the system, 1,2,4-TrCB first generated low chlorobenzene, chlorophenol, and other intermediates with simple structures and low toxicity through reactions. These substances further reacted to generate phenols and esters, and then the benzene ring broke to generate alcohols, fatty acids, etc. Finally, these simple organic substances were mineralized into CO_2_ and H_2_O. In this process, the porous carriers protected the microorganisms from the adverse effects of photocatalysis; microbial metabolism usually increases opportunities for interactions between the active groups and the substrates. In the system, the h^+^ radical contributed the most to the degradation of 1,2,4-TrCB, followed by ·OH and ·O_2_^−^. In the microbial community, the dominant bacteria were the Metallopolitalis and Rhodopseudomonas genera, which played an important role in the degradation of 1,2,4-TrCB. The ICPB provided the advantages of robust oxidability and absolute mineralization and therefore has important research value and application prospects in the removal of refractory organics.

## 4. Conclusions

(1) The ICPB system was constructed using a sugarcane cellulose-TiO_2_ carrier. This technology played an important role in promoting the degradation of 1,2,4-TrCB. Compared with biodegradation and photocatalysis alone, the removal rates of 1,2,4-TrCB increased by 68.01% and 14.81%, respectively, and the mineralization rates increased by 50.30% and 11.50%, respectively.
(2) The sugarcane cellulose-TiO_2_ carrier protected the dominant bacteria in the biofilm from damage by photocatalysis. The microorganism decomposed some of the photocatalysis products, making more free radicals that were used for the degradation of the intermediate products, thus improving the degradation rate and mineralization rate of 1,2,4-TrCB.

## Figures and Tables

**Table 1:** Intermediate products of 1,2,4-TrCB.

No.	Molecular Formula	*m*/*z*	Proposed Molecular	Proposed Structure
M1	C_6_H_14_O_2_	118.10	3-Hexyl hydroperoxide	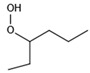
M2	C_6_H_4_Cl_2_	145.97	o-Dichlorobenzene	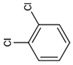
M3	C_6_H_4_Cl_2_	145.97	p-Dichlorobenzene	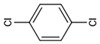
M4	C_8_H_18_O	130.14	2-Ethylhexanol	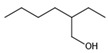
M5	C_6_H_3_Cl_3_	179.93	1,2,4-Trichlorobenzene	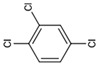
M6	C_10_H_20_O	156.15	2-Decenol	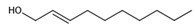
M7	C_10_H_22_O	158.17	Tetrahydrolavandulol	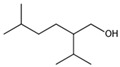
M8	C_11_H_10_O_6_	238.05	3,4-Bis (methoxycarbonyl) benzoic acid	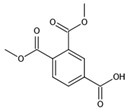
M9	C_14_H_22_O	206.17	2,4-di-Butylphenol	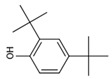
M10	C_16_H_22_O_4_	278.15	1,2-dicarboxylate -dibutyl benzene